# *Notes from the Field: Borrelia mayonii* Lyme Disease — New York, 2025

**DOI:** 10.15585/mmwr.mm7521a2

**Published:** 2026-06-04

**Authors:** Tanvir Noor Nafiz, Melissa A. Prusinski, Sai Gubbala, Jennifer White, Dowd Naik, Jamie Sommer, Danielle Wroblewski

**Affiliations:** ^1^Wadsworth Center, New York State Department of Health; ^2^Bureau of Communicable Disease Control, New York State Department of Health.

**Summary**

**What is already known about this topic?**

*Borrelia mayonii* is a pathogen that has been indicated as a causative agent of Lyme disease and identified in humans and ticks in Minnesota and Wisconsin.

**What is added by this report?**

In July 2025, a New York resident with no reported travel received a positive *B. mayonii *test result. One *Ixodes scapularis* nymph collected from the patient’s property was positive for *B. mayonii* by novel polymerase chain reaction assay. Subsequent targeted sampling identified nine additional *B. mayonii*–positive ticks from this property, indicating local transmission.

**What are the implications for public health practice?**

These findings represent the first detection of *B. mayonii* in the state of New York. The distribution of newly emerging tickborne pathogens can be used to evaluate risk and guide targeted prevention strategies.

## Introduction

On July 8, 2025, the New York State Department of Health (NYSDOH) was notified by a commercial laboratory that a Herkimer County resident had received a positive test result via real-time polymerase chain reaction (PCR) testing for *Borrelia mayonii,* a less common bacterial cause of Lyme disease than *B. burgdorferi *(Lyme disease bacterium); *B. mayonii* is transmitted by blacklegged ticks (*Ixodes scapularis*) and has previously been reported only in Minnesota and Wisconsin (*1*). The patient sought medical care with onset of symptoms consistent with a tickborne infection in late June and was coinfected with *Anaplasma phagocytophilum* (anaplasmosis bacterium). The patient was treated with doxycycline and made a full recovery (Treatment of Anaplasmosis | Anaplasmosis | CDC). The patient had spent time outdoors but had no history of travel, transplant, or transfusion, prompting a public health investigation to determine the etiology of the *B. mayonii* infection.

## Investigation and Outcomes

On July 22, 2025, NYSDOH Vector Ecology Laboratory staff members collected 147 *I. scapularis* nymphs along hiking trails through the wooded property surrounding the residence of the patient with *B. mayonii* infection and 22 from a nearby forest. Ticks were tested for *B. mayonii* using a real-time PCR assay based on previously published primers and probe and validated by the Wadsworth Center Bacteriology Laboratory to detect *B. mayonii* in ticks collected during NYSDOH’s routine pathogen surveillance and case investigation efforts (*2*)*.* The limit of detection for this assay was 34 genome copies for *B. mayonii* with 100% specificity. This project represents public health practice by NYSDOH, and institutional review board review was not required

One nymph (0.7%) from the patient’s yard was positive for *B. mayonii*. The positive nymph was co-infected with *B. burgdorferi* and *A. phagocytophilum*. NYSDOH investigators returned on October 29, 2025, and collected 305 adult ticks from these two locations, detecting nine additional *B. mayonii*–positive ticks from the patient’s property (nine of 229; 3.9%), four of which were co-infected with *B. burgdorferi.* To determine whether *B. mayonii*–positive ticks were present elsewhere in New York or before this investigation, 1,309 additional ticks collected during 2021–2025 from 23 other New York counties (1,044 collected and tested for *B. mayonii* by NYSDOH using the assay described previously, and 265 collected by NYSDOH and screened for pathogenic *Borrelia* species, including *B. mayonii*, by CDC using the assay described as part of a separate investigation) (*3*); all were negative for *B. mayonii*. Overall, 1,518 individual ticks, (1,437 *I. scapularis* and 81 *Dermacentor variabilis* [American dog ticks]), collected by standardized dragging surveys from 24 New York counties were screened by NYSDOH (Figure), including 474 *I. scapularis* from the patient’s residence and nearby forest collected as part of this investigation. The only ticks that tested positive for *B. mayonii* were collected in 2025 from the Herkimer County case property.

**FIGURE:**
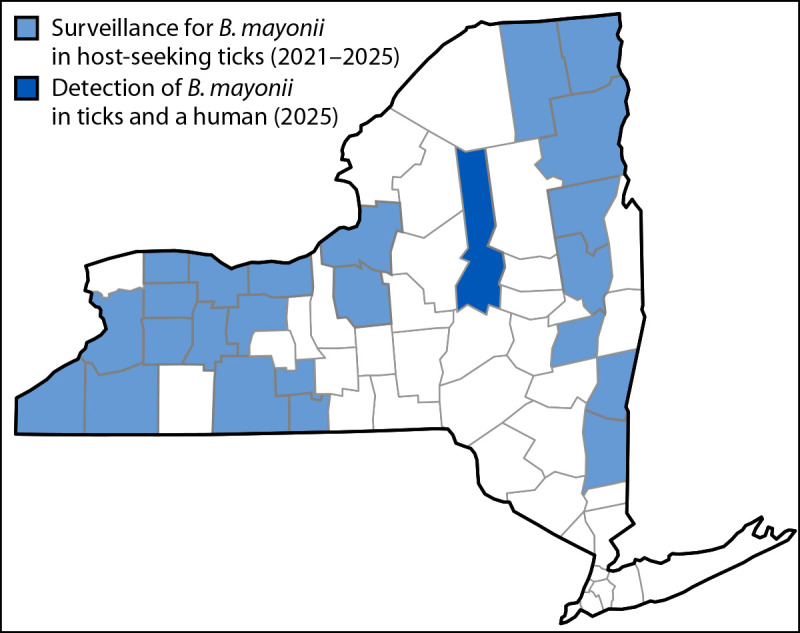
*Borrelia mayonii* surveillance and detections, by county — New York, 2021–2025

## Preliminary Findings and Conclusions

The findings of this investigation indicate local peridomestic tickborne transmission of *B. mayonii*. The overall prevalence of *B. mayonii* among nymphs and adults tested by NYSDOH statewide during the study period was 0.2% (one of 627) and 1.0% (nine of 891), respectively. The higher prevalence of *B. mayonii* observed in adult ticks (3.9%) compared with nymphs (0.7%) collected from the same location (the patient’s property) and year suggests the presence of a competent local vertebrate reservoir, such as mice or squirrels, and an established focus of enzootic transmission as opposed to incidental introductions of bird-dispersed infected immature ticks originating from the Midwest.

This study provides the first evidence of *B. mayonii* presence in New York ticks and locally acquired *B. mayonii* infection in a New York resident. Further characterization using next-generation sequencing is necessary to assess the genomic relatedness of New York *B. mayonii* isolates to the original reference strain. Continued entomological, molecular, and human tickborne disease surveillance are critical for understanding the distribution and public health significance of emerging tickborne pathogens in New York. 
